# Predicting Outcomes of Prostate Cancer Immunotherapy by Personalized Mathematical Models

**DOI:** 10.1371/journal.pone.0015482

**Published:** 2010-12-08

**Authors:** Natalie Kronik, Yuri Kogan, Moran Elishmereni, Karin Halevi-Tobias, Stanimir Vuk-Pavlović, Zvia Agur

**Affiliations:** 1 Institute for Medical BioMathematics, Bene Ataroth, Israel; 2 College of Medicine, Mayo Clinic, Rochester, Minnesota, United States of America; University of Muenster, Germany

## Abstract

**Background:**

Therapeutic vaccination against disseminated prostate cancer (PCa) is partially effective in some PCa patients. We hypothesized that the efficacy of treatment will be enhanced by individualized vaccination regimens tailored by simple mathematical models.

**Methodology/Principal Findings:**

We developed a general mathematical model encompassing the basic interactions of a vaccine, immune system and PCa cells, and validated it by the results of a clinical trial testing an allogeneic PCa whole-cell vaccine. For model validation in the absence of any other pertinent marker, we used the clinically measured changes in prostate-specific antigen (PSA) levels as a correlate of tumor burden. Up to 26 PSA levels measured per patient were divided into each patient's training set and his validation set. The training set, used for model personalization, contained the patient's initial sequence of PSA levels; the validation set contained his subsequent PSA data points. Personalized models were simulated to predict changes in tumor burden and PSA levels and predictions were compared to the validation set. The model accurately predicted PSA levels over the entire measured period in 12 of the 15 vaccination-responsive patients (the coefficient of determination between the predicted and observed PSA values was *R* ^2^ = 0.972). The model could not account for the inconsistent changes in PSA levels in 3 of the 15 responsive patients at the end of treatment. Each validated personalized model was simulated under many hypothetical immunotherapy protocols to suggest alternative vaccination regimens. Personalized regimens predicted to enhance the effects of therapy differed among the patients.

**Conclusions/Significance:**

Using a few initial measurements, we constructed robust patient-specific models of PCa immunotherapy, which were retrospectively validated by clinical trial results. Our results emphasize the potential value and feasibility of individualized model-suggested immunotherapy protocols.

## Introduction

Prostate cancer (PCa) is the second most common malignancy in men [1]. Primary treatment includes prostatectomy and/or radiation therapy. If circulating levels of prostate-specific antigen (PSA) increase after primary therapy, they indicate activation of residual cancer that is then therapeutically controlled by androgen deprivation. However, disseminated cancer cells often become androgen-independent, leading to another increase in circulating PSA levels and manifesting metastases [1]. From the observation of the latter rise in PSA level to the appearance of symptomatic metastases, the disease does not exert symptoms affecting physical wellbeing. For this reason, no therapy is administered, lest the quality of life be adversely affected by chemotherapy that is currently used in terminal PCa [2]. Thus, the period of asymptomatic PSA level increase has been considered appropriate for studies testing the efficacy of immunotherapy that is usually devoid of major adverse events.

PCa immunotherapy has begun to yield encouraging clinical effects, though not a definitive cure [3]–[4]. For example, partial responses have been induced by autologous transfer of *ex vivo* activated antigen presenting cells [5]–[6], cytokine-secreting tumor vaccines [7], vaccines containing recombinant proteins or nucleic acids and other cell-based strategies targeting cancer antigens, such as PSA or prostate-specific membrane antigen [8]. Most recently, a treatment employing *ex vivo* processed autologous antigen presenting cells combined with prostatic acid phosphatase [9] has received regulatory approval for treatment of metastatic PCa. In a recent phase 2 clinical study, an allogeneic PCa whole-cell vaccine stimulated expansion of tumor-specific immune cells in non-metastatic androgen-independent PCa patients [10]. The treatment was safe, and the rate of PSA increase (“PSA velocity”) was reduced in 11 out of the 26 studied patients [10]. Yet, the patients demonstrated a significant variability in response to treatment, that could be due to differences in individual immune history and tumor biology [11]. Suppressed immunity in PCa patients could also contribute to the relative lack of efficacy of PCa immunotherapy [12]–[16]. Restoring and enhancing immunity should be a major goal of immunotherapy [17], yet the complexity of immune system defies the attempts to achieve it. For that reason, immunity has been often studied by mathematical modeling.

Mathematical modeling has been a valuable tool in describing, quantifying and predicting the behavior of complex systems. In particular, mathematical models have played an important role in providing non-intuitive insights into tumor growth and progression [18]–[21], tumor-associated angiogenesis [22]–[25], and evolution of drug resistance [26]–[27]. Mathematical models have been successfully validated and applied for rational design of cancer therapy, for optimizing efficacy while minimizing toxicity [28]–[32], and for streamlining drug discovery and development [33]. More recently, cytokine-based and cellular immunotherapy have been modeled and scrutinized [34]–[44], and some models were validated experimentally and clinically [39], [45].

Differences in individual responses to PCa vaccination [10] raise the question whether mathematical modeling can aid in predicting the effects of immunotherapy on a single patient by quantitatively describing the interactions of cancer and the immunotherapy-modulated immune system. To study this question, we have developed a simple mathematical model describing the basic time-dependent relationships of PSA and immunity in patients treated by the allogeneic PCa whole-cell vaccine [10]. The PSA levels measured for each patient [10] were used to individualize and validate our model. Although PSA has been abandoned as a quantitative measure of PCa [46], in the absence of a more pertinent marker we used its circulating levels as a correlate of tumor burden and indicator of acute perturbation by therapy. By simulating therapy outcomes following *in silico* treatment modification (adjustment of the vaccine dose or administration schedule), we have also defined the individualized treatment protocols to be tested for more effective clinical outcomes.

## Results

### General mathematical model

**Figure 1:**
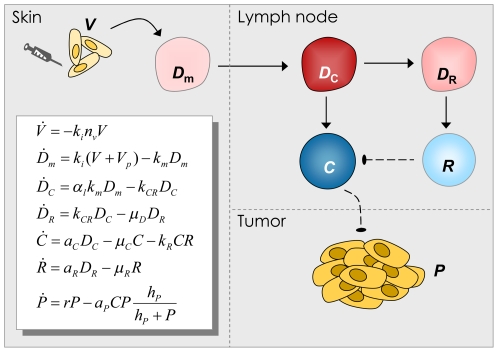
Model of interactions among the cellular vaccine (*V*), immune system and prostate cancer cells (*P*). *D_m_*, antigen-presenting dermal dendritic cells; *D_C_*, mature dendritic cells; *D_R_*, “exhausted” dendritic cells; *R*, regulatory/inhibitory cells; *C*, antigen-specific effector cells (*e.g*., cytotoxic T cells).

First, we constructed a general mathematical model of the immune response in PCa patients receiving vaccination therapy (Fig. 1, Methods and Supplemental Material S1). The model gives a general description of the dynamics of the disease, immune stimulation and immune suppression. It takes into account the time-dependent interplay of these processes, as affected by ongoing vaccination, all determining the ultimate clinical outcome. The model can be individualized by patient-specific parameters.

### Retrospective model validation

**Figure 2:**
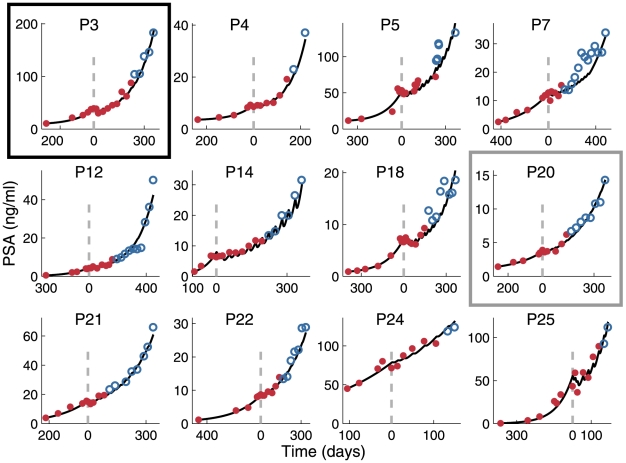
Validation of individualized models for patients responding to vaccination. Patient-specific best-fit model parameters were derived by fitting the model to the respective pretreatment PSA values and the initial in-treatment PSA values (red). Subsequent PSA levels (blue) were predicted by the use of the obtained best-fit parameters. In this and subsequent figures vertical dashed lines indicate the beginning of vaccination treatment on day 0. Achieving good predictive power required a different size of the training set for each patient. The black box emphasizes Patient 3 whose data are analyzed in Fig. 3A; the gray box pertains to Patient 20 analyzed in Fig. 3B.

Next, we tested the ability of the model to describe the PSA course in the patients who initially responded to therapy (see Methods). We used the PSA levels measured before and during the initial five to nine treatment cycles (the total of 10–15 measurements; “training set”) to individualize the model. Individual models successfully predicted the PSA course during the subsequent cycles and beyond (“validation set”) in 12 among 15 responders (Fig. 2). The predicted PSA values conformed closely to the measured values in the validation sets (*R* ^2^ = 0.972).

**Figure 3:**
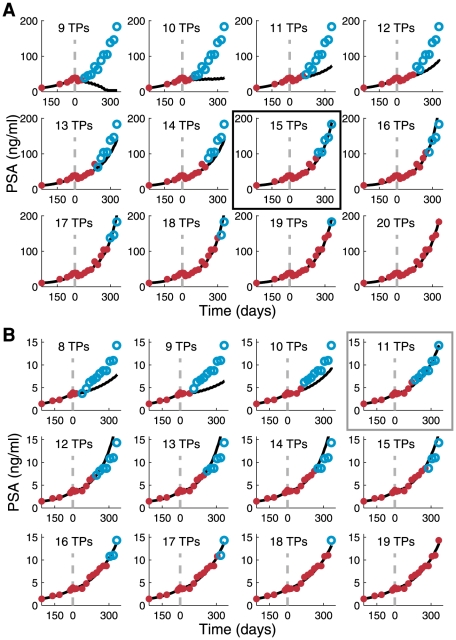
Model calibration by increasing the size of the training set. The number of PSA measurements in the training sets (red) for Patient 3 (A) and Patient 20 (B) was gradually increased, and the fitted model predictions were compared to the PSA measurements in the validation set (blue). TPs  =  training points. Boxed panels (15 TPs in A, 11 TPs in B) indicate the individually adjusted, minimal training sets that yield accurate model predictions (also shown in boxed panels in Fig. 2).

The initial stepwise increase of the size of the training set improved the prediction accuracy for all patients, but at some point the improvement became negligible (Fig. 3). Prediction accuracy as a function of the training set size followed different patterns in different patients. For example, for Patient 3, the prediction accuracy improved gradually and monotonically to reach the near-best level with rather few training points (Fig. 3A, seventh panel). In contrast, for Patient 20, a good accuracy was achieved already at the fourth iteration with 11 training points (Fig. 3B, fourth panel), but with more training points the accuracy lessened until it stabilized at iteration 9.

**Figure 4:**
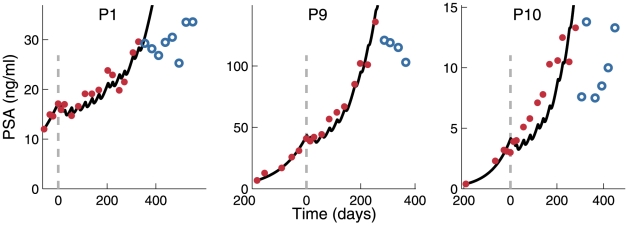
Validation of individualized models for patients with non-monotonous PSA course. Best-fit model parameters for Patients 1, 9, and 10 were obtained by fitting the model to the training set (red). Solid lines indicate the predicted subsequent directions of the PSA level change. However, the measured PSA values indicate a drastic change in the behavior of PSA levels (blue).

Three patients displayed unusual and inexplicably abrupt changes in PSA levels, or inconsistent PSA trends, towards the end of treatment; the model could not account for this behavior (Fig. 4). However, for these patients the overall fit during most of the vaccination treatment was in good agreement with PSA values.

### Personalizing model-guided therapy

Having validated the model, we could test the response to modification of treatment, *i.e*., to the change in dose size or administration schedule. We hypothesized that by the use of personalized models, we can suggest treatment modifications to stabilize PSA levels. Consequently, we simulated treatment protocols modified either by an increased vaccine dose or decreased intervals between vaccinations in the individually parameterized models, for the nine patients who completed treatment.

We found that for each patient, the putative stabilization of PSA levels required different modifications of vaccine dose or interval between vaccinations (Table 1). For example, for Patient 14 a moderate reduction of the interval (*i.e*., 21 days compared to the standard 28 days) was predicted to suffice, while other patients required more frequent vaccination with the standard dose (2.4×10^7^ cells). Patient 20, however, required either the largest among the considered vaccine doses (a 30-fold increase), or daily administration of the standard dose.

**Table 1:** Individualized therapy modifications predicted to prevent tumor progression.

Patient No.	Dose increase factor	Administration interval (days)
3	2.5	15
5	2.3	17
7	3.2	16
12	2.7	16
14	1.5	21
18	2.1	18
20	27.9	1
21	5.0	12
22	5.08	12

Minimal dose increase or maximal administration interval required to prevent in-treatment PSA elevation of more than 10 percent, analyzed for patients who completed the treatment course.

**Figure 5:**
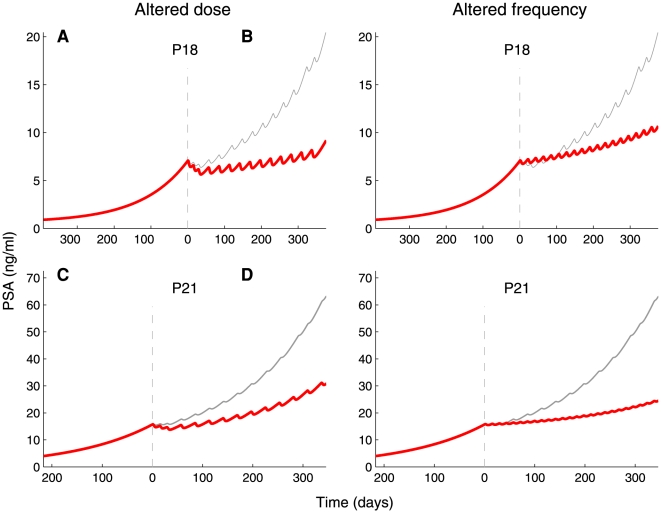
Stabilizing PSA levels by model-aided modification of the vaccination regimen. Individualized models for Patients 18 and 21 were used to predict PSA dynamics after modification of the vaccination regimen within limits deemed clinically possible. Thin gray lines represent the best-fit curves to PSA dynamics observed under the standard treatment regimen (2.4×10^7^ vaccine cells administered every 28 days; compare with Fig. 2); thick red lines are the predicted courses of PSA levels when vaccination regimens is modified. For Patient 18, the simulated effects are shown of the doubling of vaccine dose (4.8×10^7^ vaccine cells; A) or reducing the vaccination interval to 21 days (B). For Patient 21, the vaccine dose was tripled (7.2×10^7^ vaccine cells; C) or vaccination interval halved (D).

To maintain the suggested regimens within clinical constraints, we studied the effects of reducing the interval between vaccinations to 14 or 21 days, or of doubling or tripling the standard dose, and compared the predicted outcomes to the actually measured effects of the administered standard treatment. Fig. 5 displays two examples of such a comparison: for Patient 18, vaccinations with the standard dose administered more often (every 21 days), or the double dose administered at the standard 28-day interval, are predicted to yield similar effects on PSA levels. In Patient 21, the same predicted effect would be induced by the standard dose administered as frequently as every 14 days, or by the triple dose administered every 28 days.

## Discussion

Co-evolution of tumors and immunity is complex and not fully understood. The process includes time-dependent interactions that shape both immunity and the tumor, and determine which will prevail. For situations when immunity prevails, we have little information about the factors that determine the outcome; when tumors prevail, we observe only their manifest phase although tumor effects on immunity may have started even at the precancerous phase [47]. The paucity of pertinent information and the inherent complexity of the system call for mathematical modeling to formally describe and quantify the co-development of malignancy and immunity, and to predict strategies for additional immune manipulation to enhance clinical outcomes. The feasibility of this approach is rooted in the role mathematical models have played in providing non-intuitive insights into tumor growth, progression, and treatment.

We have developed a simple mathematical model, individualized it by fitting to PSA values recorded in individual patients before and during vaccination therapy, validated the model by subsequent individual PSA values, and used the results to predict the immediate response of PSA levels to modifications of vaccine dose or administration schedule. The model was remarkably successful in predicting PSA level changes in 12 out of 15 analyzed treatment-responsive patients. The manifested robustness of the fits was not compromised by the model simplicity, encompassing no more than four patient-specific parameters, with other parameters being derived from preclinical and clinical information collected from disparate published sources. Apparently, a generic representation of the interplay of immune activation and suppression suffices to describe clinical responses without the need to consider all individual mechanistic elements participating in immune regulation separately.

Derivation of patient-specific parameters from training sets and the successful validation of individualized models ascertain the predictive power of our model. For three patients, validation was unsuccessful because of the non-monotonous behavior of PSA levels at the end of vaccination course. Of note, deviation of the course of PSA levels from monotony could indicate unpredicted significant changes in the dynamic relationships between immunity and the tumor. It is tempting to speculate that this took place because vaccination broke down tumor progression.

As responses to vaccination differed among the patients significantly, a major motive for this study was to ascertain the feasibility of improving individualized treatment. Having validated the individually parameterized models of the effect of vaccination, we tested whether the model can suggest modifications in vaccine dose or administration schedule needed to stabilize PSA levels. The suggested changes also differed among patients, a finding emphasizing the potential value of testing individualized vaccination protocols in clinical trials. It is noteworthy that modifications of either the size of vaccine dose or the interval between doses could result in comparable tumor responses, allowing considerable flexibility in the choice of clinically and logistically most feasible protocols. Thus, the benefit of the method is that it could identify the patients who will not respond to therapy and enhance treatment efficacy for those who will.

To obtain an accurate predictive model, we found that each patient required a different number of PSA measurements to complete his personal training set. This raises the question whether one can determine, during treatment, the number of measurements sufficient for evaluation of personal model parameters, so that the model can be used for individualized modification of the subsequent course of treatment. Hence, we recently developed an algorithm by which one can determine the number of accumulated measurements that suffices for completion of the training set (Y. Kogan *et al*., in preparation).

The mechanistic underpinning of the model could be greatly enhanced by the introduction of factors directly related to changes in frequency and activity of immune cells and molecules, their integrated effects on the tumor, as well as the effects of the tumor on immunity. This, however, is a formidable task, as demonstrated by numerous laboratory parameters compiled from the same patients we analyzed in this study [10]. In our analysis, no single immune parameter correlated with the delay of the onset of PSA progression (an endpoint of the study); however, an artificial intelligence analysis uncovered tenuous trends in integrated outcomes of multiple parameters that might drive immunity into a particular direction (*e.g*., towards T_H_1-type response [10]). This insight opens numerous possibilities, however challenging, for constructing and testing deterministic mathematical models of co-evolution of tumors and immunity and studying the role of immune manipulation for therapeutic purposes. Our model can be employed with other biomarkers of tumor progression or other treatment types. Its use for other cancer indications should be examined.

In summary, we have presented and retrospectively validated a novel personalized mathematical model of short-term effects of vaccination on PCa. By iterative model fitting, we discovered that only a few pretreatment and in-treatment PSA measurements suffice to produce a predictive personalized model. The possibility to anticipate clinical outcomes before completion of treatment opens the door to in-treatment therapy modification to enhance the clinical response.

## Materials and Methods

### Patients and treatment

We collected the de-identified data from a Phase 2 clinical trial of an *in vitro* prepared allogeneic PCa whole-cell vaccine, administered to asymptomatic nonmetastatic PCa patients, whose circulating PSA levels were rising despite androgen suppression (Cohort 1 in ref. [10]). Treatment included 14 intradermal applications of the vaccine, the first two containing *Bacille Calmette-Guérin* (BCG). The initial three doses were spaced two weeks apart, followed by 11 doses spaced four weeks apart, so that the treatment period lasted approximately one year. Circulating PSA was measured prior to treatment, at vaccine injections, and sporadically between injections and after completion of treatment. The number of pre-treatment, in-treatment and post-treatment PSA measurements varied among the patients.

To classify the patients by response to treatment, we evaluated individual PSA velocity (linear change of logarithm of PSA levels) before therapy and during the first four cycles of therapy. In 15 patients vaccination reduced the PSA velocity, while in others it did not. We studied the PSA data solely from the responding patients.

### General mathematical model

We modeled the basic interactions of PCa and immunity by a system of seven ordinary differential equations accounting for interactions of the vaccine, the immune system and cancer cells within the skin, the lymph nodes and other tissues (disseminated tumor cells). The model is based on the assumption that the vaccine stimulates cancer-specific immunity, but also that normal regulatory mechanisms and the tumor suppress this immunity. The model is fully detailed in Fig. 1 and Supplemental Material S1.

### Model implementation

The model and curve-fitting algorithms were implemented on a MATLAB® programming platform (MathWorks, Natick, MA). We solved the model equations by the numerical ordinary differential equation solvers available in MATLAB. Fitting of the model to the data was performed using constrained optimization procedures.

### Individualized models

To individualize the model, we evaluated selected parameters for individual patients. Most model parameters were evaluated from published *in vitro* and *in vivo* data and were assumed to be similar for all patients (Table 2; for details, see Supplemental Material S1). Tumor growth rate (*r*), CTL killing activity (*a_p_*), as well as *A* and *B*, the two parameters correlating tumor burden and PSA levels, were considered patient-specific, based on the observations that tumor growth rates, PSA secretion rates and intensity of vaccine-induced immune response vary significantly among individuals. To avoid over-parametrization, we attributed the intensity of immune response to the single parameter *a_p_*.

**Table 2:** Model parameters.

Parameter	Definition^a^	Value	Units	References
*k_i_*	Rate of DC maturation following vaccine uptake	0.06	h^−1^	[48]
*n_V_*	Number of vaccine cells required to induce maturation of one DC	1	–	[49]–[51]
*V_p_*	Natural influx of mature DCs	0	cells	Estimate
*k_m_*	Rate of DC migration from skin to lymph node	0.027	h^−1^	[52]
*α_l_*	Fraction of antigen-presenting DCs entering the lymph node	0.03	–	[52]
*k_CR_*	Rate of exhaustion of mature DCs	0.027	h^−1^	[53]
*µ_D_*	Death rate of exhausted DCs	0.014	h^−1^	[54]
*a_R_*	Rate of inhibitory cell recruitment by exhausted DCs	3×10^−3^	h^−1^	[55]
*µ_R_*	Death rate of inhibitory cells	0.03	h^−1^	[56]
*a_C_*	Rate of effector cell recruitment by mature DCs	0.38	h^−1^	[49]
*µ_C_*	Effector cell death rate	0.007	h^−1^	[41]
*k_R_*	Rate of effector cell inactivation by inhibitory cells	6×10^−7^	cell^−1^×h^−1^	[57]
*r*	Tumor growth rate	Patient specific	h^−1^	-
*a_p_*	Maximal PCa cell killing efficacy	Patient specific	cell^−1^×h^−1^	[58]–[59]
*h_P_*	Effector cell efficacy damping coefficient	10^8^	cells	[58]–[59]

Definitions, values and units of parameters and their sources of estimation are indicated. The full parameter evaluation process is explicated in Supplemental Material S1, Section B.

a Abbreviations: DCs, dendritic cells; PCa, prostate cancer.

To estimate patient-specific parameters in individual models, we fitted the model by the least-squares method to the pertinent “training set” that included all pre-treatment and several initial in-treatment PSA values for each patient; the number of training data points could differ among the patients. Next, we used the results to simulate the subsequent course of PSA change and compared the simulation with PSA measurements recorded following measurements in the training set (“validation set”). If prediction accuracy was low, the size of the training set was iteratively increased by a subsequent PSA measurement, subtracting the point from the validation set.

### Model validation

To predict PSA dynamics beyond the training set, we simulated each individualized model under the personal vaccination schedule (which could include minute variations from the general schedule). For each patient, predictions were compared with the clinically observed PSA levels in the validation set. Goodness-of-fit was evaluated pooling together all the validation data points from all the patients. To compare predictions with measurements, we calculated the coefficient of determination, *R* ^2^, between the predicted and observed PSA values.

### Therapy individualization

We probed whether intensifying treatment could improve vaccine efficacy in individual patients. Hence, based on the validated individual models for the nine patients who completed treatment, we simulated the effects of many intensified vaccination protocols for each patient. Intensification included a graded 10 percent increase above the standard vaccine dose or graded one-day reduction of administration interval relative to standard schedule. We singled out individual vaccine administration schedules that should lead to stabilization of PSA levels at the end of treatment, at concentrations not more than 10 percent above the pre-treatment level. For each patient, minimal increase in vaccine dose and minimal reduction in dosing interval that meet the above PSA stabilization criterion are reported in Table 1.

## Supporting Information

Material S1 This file includes (a) the mathematical model of prostate cancer therapeutic vaccination; (b) parameter estimation.
(DOCX)
