# Supplementary material for: Predicting Outcomes of Prostate Cancer Immunotherapy by Personalized Mathematical Models
Source: PLoS One. 2010 Dec 8;5(12):e15482. doi: 10.1371/journal.pone.0015482 (PMC2999571; doi:10.1371/journal.pone.0015482)
Supplement: Material S1 — This file includes (a) the mathematical model of prostate cancer therapeutic vaccination; (b) parameter estimation. (DOCX) [file pone.0015482.s001.docx]

**Supplemental Material S1 to**

**Predicting Outcomes of Prostate Cancer Immunotherapy by Personalized Mathematical Models**

Natalie Kronik, Yuri Kogan, Moran Elishmereni, Karin Halevi–Tobias,

Stanimir Vuk‑Pavlović, Zvia Agur

**A. Mathematical model of prostate cancer therapeutic vaccination**

The mathematical model (Fig. 1) assumes that the vaccine, *V*, is injected into the dermis where it stimulates maturation of naïve sentinel DC precursors into mature antigen-presenting DCs, *D*_m_, at the rate *k_i_* [1, 2]. The model takes into account the additional contribution of endogenous tumor antigens at a constant level, *V*_p_. Both *V* and *V_p_* are measured in the units equivalent to tumor cells comprising the vaccine. Each DC takes up an amount of vaccine, *n*_V_, during maturation. The number of antigen-presenting DCs that migrate from skin into the lymph node is linearly proportional to the total number of mature antigen-presenting DCs in the skin, *D*_m_ (all cell populations are measured in the units of cell numbers). Time–dependent changes in *V* and *D*_m_ are given by

Antigen-presenting DCs migrate from the skin into lymph nodes at the rate *k_m_* with the probability *α_l_* to join the pool of functional antigen-presenting DCs, *D*_C_. After interaction with effector cells, functional antigen-presenting DCs become “exhausted” [3] (at the rate *k_CR_*), giving rise to a regulatory DC population, *D_R_*, [4] that dies at the constant rate *μ_D_*. Accordingly, the dynamics of the DC populations in the lymph node is described by

Functional antigen-presenting DCs induce the T_H_1-type immunity that includes recruiting and activating tumor-specific cytotoxic T lymphocytes (CTLs), *C*, responsible for killing tumor cells. Functional antigen-presenting DCs recruit CTLs [5] at the rate *a_C_*. CTLs die at the rate *μ_C_*, or are inactivated at the rate *k_R_* by regulatory/inhibitory cells, *R* [6]. Hence, the dynamics of *C* is formulated by

The population of regulatory/inhibitory cells, *R*, includes the T_reg_ cells that inhibit CTLs [7], cells expressing immunosuppressive cytokines [3, 6], and also myeloid-derived suppressor cells (MDSCs) that themselves express T_H_2 cytokines, *e.g*., IL-10 and TGF-β [8]. The existence of regulatory cells cannot be ignored, as increased amounts of regulatory CD4^+^CD25^high^ T cells (T_reg_) were detected in the blood and tumor tissue of early stage PCa patients [9]. Likewise, high levels of circulating CD14^+^HLA-DR^low/–^ cells, with *in vitro* characteristics akin to MDSCs, have also been detected in PCa [8]. We further postulate that regulatory cells are recruited by regulatory DCs at the rate *a_R_* and are eliminated at the rate *μ_R_*:

PCa cell population, *P*, is assumed to propagate exponentially, by the rate *r*. The rate of tumor-cell killing is assumed to be proportional to CTL numbers, with the coefficient *a_P_*. Tumor killing efficacy decreases with increasing tumor burden, as reflected by the value of parameter *h_P_* (for derivation of this expression see ref. 10). Thus, the tumor dynamics is described by

Vaccine treatment was modeled as instantaneous additions to *V(t)* of the number of vaccine cells given at each injection time for a specific patient. We have implemented individual schedules available from the personal clinical files.

**B. Estimating parameter values**

We estimated the values of model parameters from the literature:

***k_i_,* the rate of vaccine uptake.** We assumed that the uptake of the cellular PCa vaccine is similar to the uptake of a microglia (MCG) cellular vaccine into rat brain [11]. In a preclinical study of brain cancer immunotherapy, three and five days after injection of labeled MCG cells, one could still detect 17.5 percent and 3.5 percent of labeled cells, respectively [11]. We describe these data by

, ;

where *V* is vaccine cell number*, D* the labeled MCG cell number, *k_i_* the parameter to be estimated and *μ* the rate of labeled cell loss (*e.g*., by migration). The initial data for the system are *V*(0)=1×10^6^ cells (two infusions of 5×10^5^), *D*(0) *=* 0.

To translate the measured percentages into cell numbers, we had to estimate the total number of MCG cells in brain. Between 50,000-400,000 MCG cells were isolated from the rat brain in [11], yet this is likely an underestimate of actual cell counts due to tissue degradation that lead to cell loss. In a study of murine brain, roughly 500,000 MCG cells were obtained from the two hippocampi [12]. Considering that murine hippocampi constitute roughly 5 percent of the total brain [13], and assuming that the same relation holds for the rat and that the MCG density in other parts of the brain is similar to the hippocampus, we evaluate a total of 1×10^7^ MCG cells. Thus, we estimate that the rat brain contains 5×10^6^ MCG cells, an average of the two estimates. Based on this, we assessed that 875,000 MCG cells were present in the rat brain on day 3 and 175,000 cells on day 5. Using the analytic solution of the above system of two equations, by least squares approximation we found that *k_i_* ≈ 0.0674 *h*^–1^. However, the fit to data points was not very accurate due to the large difference between the two measurements, which forced high values of *μ* and *k_i_*; the latter value, in turn, requires that the exhaustion rate of the vaccine is faster than the measured values permit. To increase the accuracy of the fit, we allowed the total MCG cell estimate in rat brain to vary between 1×10^6^ and 1×10^7^ cells, while fitting the parameters. Under these conditions, our best estimate was $k_{i}\approx0.06\mathop{}\limits h^{-1}$.

***n_V_*, the number of vaccine cells taken up by a dendritic cell.** Optimized antigen presentation required the ratio of one dead tumor cell (as antigen) and one immature DC [14]. Consequently, we assumed *n_V_*≤1, *i.e*., that each vaccine cell activates at least one DC. As the vaccine is comprised of whole cells, we surmise that on average one DC consumes one vaccine cell; thus, *n_V_* = 1.

***V_p_*, role of antigens from the tumor *in situ*.** This parameter reflects the innate antigenicity of the tumor that allows for constant recruitment of DCs. The parameter is measured in the units of *V* and, hence, cannot be estimated from the literature or by direct measurement. However, it can be derived indirectly by model fitting to patient data. We set its value to zero, assuming strong immune suppression by the tumor.

***α_l_*, probability of mature dendritic cells reaching lymph nodes; *k_m_*, migration rate of mature dendritic cells into lymph nodes.** After mature DCs had been injected into footpads of mice, between 0.01 percent and 3 percent of injected cells were found in the lymph nodes 48 hours later (Fig. 1A, B in ref. 15). As we posit that *α_l_* reflects the overall probability of a DC reaching a lymph node, we conclude *α_l_* ≈ 0.03. To interpret the time course data from [15], we described the cell dynamics by

, ;

where *D_m_* is the number of injected DCs and *D_C_* the number of DCs that reached a lymph node. The magnitude of *α_l_* and *k_m_* are to be determined, while *μ* stands for the loss of DCs from the lymph node. The initial data are given by experimental conditions at *t* = 0. We have also considered the possibility that the DCs do not start migrating immediately after injection, but only after the delay *τ*. Consequently, we derived the best-fit parameter values for *k­_m_*, *μ*, *α_l_* and *τ* and obtained $k_{m}\approx0.027\mathop{}\limits h^{-1}$,$\alpha_{l}\approx0.03$.

***k_CR_*, the rate of DC exhaustion.** In a study of T_H_1/T_H_2 cell ratio after *in vitro* stimulation by DCs in the presence or absence of toxic shock syndrome toxin 1 (TSST-1), the percent of activated T_H_1 diminished approximately by the factor of 3 in the course of 40 hours (Fig. 3 in ref. 4). We take this observation to indicate that the numbers of non-exhausted DCs dropped by the factor of ~3, allowing the estimate $k_{CR}\approx\ln\left(3\right)/40\approx0.027\mathop{}\limits h^{-1}$.

***μ_D_*, dying rate of exhausted DCs.** We assessed the values of *D*_m_ from a measurement of the rate of BrdU incorporation into murine splenic DCs [16]; we assume these cells are the closest to the regulatory type of DCs considered in our study. The linear decay model was fitted to the data; the model entails that the proportion of BrdU-labelled cells at time *t* is $1-e^{-\mu_{D}t}$. The resulting best–fit value was $\mu_{D}\approx0.014\mathop{}\limits h^{-1}$.

***a_R_*, rate of inhibitory cell induction by tolerogenic DCs.** We assessed this parameter from a study of the role of tolerogenic DCs in raising T_reg_ cells [17] that found that the addition of immature tolerogenic DCs increased the number of CD4^+^CD25^+^ T_reg_ cells *in vitro* by 7 percent. Under the experimental conditions in ref. 17, we calculated that each tolerogenic DC induced 0.36 T_reg_ cells in 120 hours leading to the estimate $a_{R}\approx3\times10^{-3}\mathop{}\limits h^{-1}$.

***μ_R_*, death rate of inhibitory cells.** We estimated this parameter from the turnover of human T_reg_ cells (Fig. 2, ref. 18]. Assuming no proliferation of T_reg_ cells, we fitted the linear degradation model for T_reg_ cells to data for the three subjects studied [18] and obtained estimates of *μ_R_* of 0.037 cells/h, 0.021 cells/h and 0.032 cells/h, respectively, yielding the mean value $\mu_{R}\approx0.03\mathop{}\limits h^{-1}$.

***a_C_*, the rate of CTL recruitment by mature DCs.** In a study of uptake and presentation of tumor cell lysate by myeloid DCs, equivalent numbers of T cells and mature DCs were coincubated for 14 days when the levels of CD8^+^CD25^+^ CTLs were measured [14]. At that time, CD8^+^CD25^+^ cells comprised 10 percent of T cells (ca. 2×10^5^ cells) compared to 0.08 percent in DC-free control. Assuming no CTL proliferation, we calculated the lower limit of the CTL recruitment rate $a_{C}\approx0.00298\mathop{}\limits h^{-1}$. As, however, CTLs underwent seven or more divisions in the course of experiment, multiplying the lower limit of the *a_C_* value by 2^7^, yielded the estimate of the actual *a_C_* value as 0.38 *h*^–1^.

***μ_C_*, CTL death rate.** We estimated the CTL death rate elsewhere as $\mu_{C}\approx0.007\mathop{}\limits h^{-1}$[10].

***k_R_*, rate of CTL inactivation by inhibitory cells.** We estimated the value of *k_R_* from the data in ref. 7. In that work, T_H_ cells were mixed with T_reg_ cells and proliferation of T_H_ cells was assessed by [^3^H]TdR uptake, which we assumed was in direct relation to T_H_ cell numbers. Assuming that T_H_ cells decay with the rate *k_R_*, (*i.e*., directly proportional to *R*, which is the number of T_reg_ cells) and using the T_H_ cell number at *R* = 0 as the base level, we estimated *k_R_* between 1.16×10^–6^ and 1.96×10^–6^ *cell^–1^h^–1^*. Because this estimation is sensitive to experimental cell numbers, and the numbers of T_reg_ cells and CTLs numbers in human body are several orders of magnitude larger than in this experiment, the parameter is expected to be somewhat smaller. Therefore, the value for this parameter was taken to be one half of the mean calculated result, *i.e*., 6×10^–7^ *cell^–1^h^–1^*.

***r*, the tumor growth rate.** The PSA levels in the patients did not reach saturation under most conditions [19]. Hence, under the conditions of the study, we assumed that tumor growth followed an exponential law. For each patient, the parameter *r* was derived by fitting to the measured PSA values. The values of *r* were found between 10^–5^ and 10^–3^ *h^–1^*.

***a_p_*, the maximal CTL killing efficacy and *h_P_*, the damping coefficient.** We estimated these values from ref. 20 that reported CTL–mediated lysis of LnCaP prostate cancer cells. With the numbers of CTLs and tumor cells unchanged during the experiment, the number of tumor cells is given by

where *C* is the CTL number and *P* the number of targeted PCa cells. We fitted the analytical solution of this equation to experimental data and $a_{P}\approx1.14\times10^{-6}\mathop{}\limits cells^{-1}h^{-1}$. We obtained similar parameter values using the data on CTL–mediated lysis of HeLa cells [21], $a_{P}\approx9.53\times10^{-7}\mathop{}\limits cells^{-1}h^{-1}$. Based on these considerations, we estimate the range $0\leq a_{P}\leq2\times10^{-6}\mathop{}\limits cells^{-1}h^{-1}$. The exact value of this parameter was derived individually for each patient by fitting to the measured PSA values.

Similarly to the case of *k_R_* estimation, the actual *in vivo* value of *h*_P_ depends on the order of magnitude of tumor cell number, which is different in patients and in experiments. Therefore, the value for *h_P_* was taken as 1×10^8^ cells, following ref. 10.

**References**

1. Banchereau J, Palucka AK (2005) Dendritic cells as therapeutic vaccines against cancer. Nat Rev Immunol 5: 296-306.

2. Banchereau J, Steinman RM (1998) Dendritic cells and the control of immunity. Nature 392: 245-252.

3. Kajino K, Nakamura I, Bamba H, Sawai T, Ogasawara K (2007) Involvement of IL-10 in exhaustion of myeloid dendritic cells and rescue by CD40 stimulation. Immunology 120: 28-37.

4. Langenkamp A, Messi M, Lanzavecchia A, Sallusto F (2000) Kinetics of dendritic cell activation: Impact on priming of Th1, Th2 and nonpolarized T cells. Nature Immunol 1: 311-316.

5. Janeway CA, Travers P, Walport M, Shlomchik MJ (2005) Immunobiology. New York: Garland Science Publishing.

6. Hollenbaugh JA, Dutton RW (2006) IFN-γ regulates donor CD8 T cell expansion, migration, and leads to apoptosis of cells of a solid tumor. J. Immunol 177: 3004-3011.

7. George TC, Bilsborough J, Viney JL, Norment AM (2003) High antigen dose and activated dendritic cells enable Th cells to escape regulatory T cell-mediated suppression in vitro. Eur J Immunol 33: 502-511.

8. Vuk-Pavlović S, Bulur PA, Lin Y, Qin R, Szumlanski CL, et al. (2010) Immunosuppressive CD14^+^HLA-DR^low/-^ monocytes in prostate cancer. Prostate 70: 443-455.

9. Miller AM, Lundberg K, Ozenci V, Banham AH, Hellstrom M, et al. (2006) CD4^+^CD25^high^ T cells are enriched in the tumor and peripheral blood of prostate cancer patients. J Immunol 177: 7398-7405.

10. Kronik N, Kogan Y, Vainstein V, Agur Z (2008) Improving alloreactive CTL immunotherapy for malignant gliomas using a simulation model of their interactive dynamics. Cancer Immunol Immunother 57: 425-439.

11. Kulprathipanja NV, Kruse CA (2004) Microglia phagocytose alloreactive CTL-damaged 9L gliosarcoma cells. J Neuroimmunol 153: 76-82.

12. Brewer GJ (1997) Isolation and culture of adult rat hippocampal neurons. J Neurosci Methods 71: 143-155.

13. Lu L, Airey DC, Williams RW (2001) Complex trait analysis of the hippocampus: Mapping and biometric analysis of two novel gene loci with specific effects on hippocampal structure in mice. J Neurosci 21: 3503-3514.

14. De Vleeschouwer S, Arredouani M, Ade M, Cadot P, Vermassen E, et al. (2005) Uptake and presentation of malignant glioma tumor cell lysates by monocyte-derived dendritic cells. Cancer Immunol Immunother 54: 372-382.

15. MartIn-Fontecha A, Sebastiani S, Hopken UE, Uguccioni M, Lipp M, et al. (2003) Regulation of dendritic cell migration to the draining lymph node: Impact on T lymphocyte traffic and priming. J Exp Med 198: 615-621.

16. Chen M, Huang L, Shabier Z, Wang J (2007) Regulation of the lifespan in dendritic cell subsets. Mol Immunol 44: 2558-2565.

17. Min WP, Zhou D, Ichim TE, Strejan GH, Xia X, et al. (2003) Inhibitory feedback loop between tolerogenic dendritic cells and regulatory T cells in transplant tolerance. J Immunol 170: 1304-1312.

18. Vukmanovic-Stejic M, Zhang Y, Cook JE, Fletcher JM, McQuaid A, et al. (2006) Human CD4^+^ CD25^hi^ foxp3^+^ regulatory T cells are derived by rapid turnover of memory populations in vivo. J Clin Invest 116: 2423-2433.

19. Michael A, Ball G, Quatan N, Wushishi F, Russell N, et al. (2005) Delayed disease progression after allogeneic cell vaccination in hormone-resistant prostate cancer and correlation with immunologic variables. Clin Cancer Res 11: 4469-4478.

20. Peshwa MV, Shi JD, Ruegg C, Laus R, van Schooten WC (1998) Induction of prostate tumor-specific CD8^+^ cytotoxic T-lymphocytes in vitro using antigen-presenting cells pulsed with prostatic acid phosphatase peptide. Prostate 36: 129-138.

21. Street D, Kaufmann AM, Vaughan A, Fisher SG, Hunter M, et al. (1997) Interferon-γ enhances susceptibility of cervical cancer cells to lysis by tumor-specific cytotoxic T cells. Gynecol Oncol 65: 265-272.
